# Sporadic parathyroid adenoma: an updated review of molecular genetics

**DOI:** 10.3389/fendo.2023.1180211

**Published:** 2023-05-08

**Authors:** Angeliki Chorti, Angeliki Cheva, Anthoula Chatzikyriakidou, Charoula Achilla, Kassiani Boulogeorgou, Krokou Despoina, Stefanos Milias, Thomas Zarampoukas, Theodossis Papavramidis

**Affiliations:** ^1^ 1st Propedeutic Department of Surgery, AHEPA University Hospital of Thessaloniki, Aristotle University of Thessaloniki, Thessaloniki, Greece; ^2^ Laboratory of Pathology, Faculty of Medicine, School of Health Sciences, Aristotle University, Thessaloniki, Greece; ^3^ Laboratory of Medical Biology - Genetics, Faculty of Medicine, School of Health Sciences, Aristotle University, Thessaloniki, Greece; ^4^ Minimal Invasive Endocrine Surgery Department, Kyanos Stavros, Euromedica, Thessaloniki, Greece; ^5^ Laboratory of Pathology, Interbalkan Medical Center, Thessaloniki, Greece

**Keywords:** parathyroid adenoma, parathyroid genomis, parathyroid proteomics, parathyroid adenoma tumorigenesis, parathyroid pathogenesis

## Abstract

**Introduction:**

Primary HPT (PHPT) is a common disorder, affecting approximately 1% of the general population. Parathyroid adenomas emerge as non-familial sporadic in 90% of cases. The aim of this review is to give a detailed update of molecular genetics of sporadic parathyroid adenoma reported in international literature.

**Methods:**

A bibliographic research was conducted in PubMed, Google Scholar, and Scopus.

**Results:**

Seventy-eight articles were included in our review. CaSR, MEN1, CCND1/PRAD, CDKI, angiogenic factors like VEGF, FGF, TGFβ, and IGF1, and apoptotic factors are important genes in parathyroid adenomas pathogenesis that have been established by several studies. A huge list of proteins is differently expressed in parathyroid adenomas measured by Western Blotting, MALDI/TOF, MS spectrometry, and immunohistochemistry. These proteins take part in several cell processes such as cell metabolism, cytoskeleton structural stability, cell oxidative stress regulation, cell death, transcription, translation, cell connection, and cell signaling transmission, while they can be found over- or underexpressed in abnormal tissues.

**Conclusion:**

This review gives a detailed analysis of all reported data on genomics and proteomics of parathyroid adenoma. Further studies should be applied on understanding parathyroid adenoma pathogenesis and introducing new biomarkers for early detection of primary hyperparathyroidism.

## 1 Introduction

Hyperparathyroidism (HPT) is defined as the hypersecretion of parathormone and it is classified as primary, secondary, and tertiary. Primary HPT (PHPT) is a common disorder, affecting approximately 1% of the general population. It is more frequent in women, with a women-to-men ratio of 3–4:1. PHPT results from a single parathyroid adenoma in 80%–85% of cases, multi-gland hyperplasia in 15%, and cancer in 1%. In the majority of cases, PHPT remains asymptomatic and is diagnosed incidentally during routine blood examination. In cases of symptomatic disease, musculoskeletal, urinary, gastrointestinal, neural, and cardiovascular systems are affected. Surgical treatment is the only curative treatment and is indicated in cases of symptomatic disease and in asymptomatic if at least one of these conditions is fulfilled: (a) age <50 years old; (b) serum Ca >1 mg/dl (>0.25 mmol/L); (c) T-score below -2.5 in one of the following bone regions: lumbar spine, total hip, femoral neck, or 1/3 distal radius bone or detection of asymptomatic vertebral fracture by any imaging method; (d) creatinine clearance <60 cc/min; (e) 24-h urine calcium >300 mg/d (<10 mmol/d) together with increased risk of kidney stones based on biochemical stone risk analysis, presence of asymptomatic nephrolithiasis, or nephrocalcinosis detected by imaging methods (1–4).

Parathyroid adenomas emerge as non-familial sporadic in 90% of cases or scarcely as familial- either as a manifestation of a syndrome such as the various forms of the MEN syndrome and the HPT- jaw tumor syndrome or as a non-syndromic hereditary form of PHPT, such as FIHPT, FHH, and NS-HPT (5).

The aim of this review is to give a detailed update of molecular genetics of sporadic parathyroid adenoma reported in international literature.

## 2 Methods

A bibliographic research was performed using PubMed, Google Scholar, and Scopus. The search terms employed were “genetic pathways AND sporadic parathyroid adenoma” and “molecular genetics AND sporadic parathyroid adenoma “. An ethical approval is not required because this study is a review of the existing international literature.

## 3 Review

A total of 199 were found under the term “genetic pathways AND sporadic parathyroid adenoma”, from which only 43 were relevant, whereas 159 articles were found under the term “molecular genetics AND sporadic parathyroid adenoma “, from which 65 were relevant. Duplicate results were excluded (30 articles). At last, 78 articles were included in our systematic review.

The first genetic studies on the pathogenesis of sporadic parathyroid adenoma were conducted in 1990, and until now a large amount of genes are associated with adenoma development. These genes are mainly regulators of cell cycle, growth factors, apoptosis factors, death receptors, and signal transduction molecules. A great variety of genetic mutations, especially somatic ones, have been observed such as base insertions, deletions, and substitutions (6, 7). **Table 1** shows genetic alterations in parathyroid adenoma pathogenesis.

**Table 1:** Proteins expressed in Parathyroid Adenomas.

PTH	CHD3	AINX	MAP3K1
QCR1	CLSPN	GSTO1	MAP3K13
ATPA	STAG2	PURA	MAP3K5
ECHM	COL14A1	IF4A3	PRDX2
FUMH	MSH6	AL1A1	PIK3CA
ATP5H	DNM2	SERA	ATP12A
CH60	DTNBP1	ANXA2	PCDHB12
IDH3A	TOPORS	AMPL	RALGAPA1
ETFA	EPHA2	IDHC	RAPGEF2
HNRNPAB	EIF3C	GBB2	RASEF
MDH2	CASR	ANXA4	RASGRF1
SODM	FGFR4	ERP29	PTPRK
ODPB	FGD6	RUVB1	PLA2R1
GPDM	KAT6A	PGM1	SLC20A2
ATPB	LAMC1	LMNA	SMC1B
CAPG	LRRC7	AGRN	SMARCC2
PRDX6	PPFIA1	TECTA	TBX2
ENOA	LRP8	APP	TNS1
GSTP1	KDM2A	AKNA	SUPT5H
TPIS	MPDU1	ABCA3	TAF3
TGM2	MADD	BRCA1	PROS1
UCHL1	MAP2	CAMSAP3	SLC30A8
K2C8	MAST1	CP	VDAC1
K1C19	MAPK8	COX5A	NUFIP1
KMT5A	UBE2N	UQCRC1	KRBOX4
NPIPA7	HNRNPA2B1	BMP2K	HSP90B1
SLTM	RPSA	MED12	HSPD1
S100A11	CALM1	LUC7L	PRDX3
PA2G4	ANXA5	TCHP	PRDX1
PARK7	MAPK1	GOLGA8Q	FGG
BSPRY	DEGS1	CCDC174	GAPDH
YWHAZ	TMBIM6	ZNF674	APOA1
CCT5	SSBP3	CTTNBP2	S100A13
SNORA74A	FZD6	ARIH2	KRT4
DPYD	ATP6AP2	GOLGA8O	EZR
ALG5	PIGG	PPM1B	PVALB
ZNF552	UBAP1	ZNF605	ALB
NBEAL1	ST13	COPS7B	TF
OGN	YTHDC2	ZNF33A	SPDYE16
CALR	CPE	NPM	PITPNB
PRDX2	MED26	ACO2	ZNF394
BCL2L10	FSD1	TUFM	RAB7B
DNAJC2	VMP1	MPP11	PHB
ATP5H	NRBF2		

### 3.1 Genomics

Alteration in CaSR (calcium sensing receptor) signaling pathway, regulating parathormone (PTH) secretion, has been identified. CaSR protein is a plasma membrane G-protein and acts as a receptor on chief parathyroid cell surface. It binds serum calcium and activates the signaling pathway, which includes the phospholipase Cβ and the protein kinase C, resulting in suppression of PTH secretion. In addition, vitamin D receptors are proliferated, binding 1.25 dihydroxivitamin D and also suppressing PTH secretion. CaSR gene, located in chromosome 33q13.3–21, spans about 103 kb and has eight exons. Exons 2–7 encode a CASR protein of 1,078 amino acids (8). It is an oncosuppressor as when suppressed, cell growth is enhanced (9, 10). CaSR gene plays an important role in parathyroid adenoma development, as only a single genetic mutations has been identified, which is not confirmed by other studies (11). In Arnold et al. studies in rats, CaSR, VDR, and RET gene evolvement in parathyroid adenoma has not been confirmed, although these are involved in hereditary disease and play significant role in parathyroid gland biological processes (12).

MEN1 gene, which plays an important role in hereditary disease, is an oncosuppressor gene, located in chromosome 11q13 and aims menin protein through Wnt/β-catenin signaling pathway (5, 10, 13). Menin acts as transcription regulator factor (12). In sporadic disease, somatic mutations in both alleles are required, which occur in 20.2%. Somatic mutations include deletions, loss, insertions, transposition, and loss of heterozygosity. Loss of heterozygosity occurs in 30.5% (11). Loss of 11q, 1p, 6q, 9p, 11p, 13q, and 15q and insertion of 7, 16p, and 19p are involved in MEN1 dysregulation. Loss of one MEN1 allele is found in 25%–40% of parathyroid adenomas, together with inactivating mutations of other alleles, which is an early event as it is identified in cases of small adenomas with mild hypercalcemia (14). MEN1 gene mutations are responsible for 15%–20% of parathyroid adenoma development (12).

CCND1/PRAD1 gene is a proto-oncogene, located in 11q13 chromosome, nearby another gene that regulates PTH secretion, and is translated to cyclin D1 protein. Cyclin D1 is a regulator factor of G1-S cell cycle phase and binds to CDK4 and CDK6 which belongs to the same family (11, 13). CCND1 gene aims the primer of PTH gene (12). Its involvement in parathyroid adenoma development is 20%–40%, whereas mutation that occurs in 8% DNA translocation is a type of mutation which occurs in 8% and leads to over expression of cyclin D1 (11, 12, 14). Overexpression of cyclin D1 has been correlated to enhancement of parathyroid cell proliferation and PTH oversecretion (12).

Mutations in CDKI genes (cyclin D1 inhibitors) are involved in CCND1 gene regulation. CDKN1B, CDKN1A, CDKN2B, and CDKN2C germline mutations have been recorded (5). Germline mutations are rare in sporadic parathyroid adenomas without familiar history. Somatic mutations on CDKNB1 gene, encoding p27kit1 protein, have been found after DNA sequencing in 86 patients. These mutations include alterations in both alleles or loss of heterozygosity and are rare (1%) (11). The result of these mutations is p27kit1 downregulation or destabilization (15). CDKN2A, CDKN2B, and RASSF1A gene expression is lower in parathyroid adenomas compared to normal tissue, caused by hypermethylation of genes’ primers in pathological tissue (16, 17). One single somatic mutation related to parathyroid adenoma has been recorded in CDKN2C gene (11).

Duan et al. found mutations related to parathyroid adenoma development in 5% of ZFX gene aiming cyclin D1, in 2%–5% of CTNNB1 gene, encoding β-catenin, in 1% of EZH2 gene, accelerating β-catenin and <1% of POT1 which is a genome stabilizer (10).

EZH2 gene encodes histone methylase H3K27 and a rare mutation (p. Y641N) has been correlated to parathyroid adenoma development (14). Gene oncogenesis mechanism has not been yet clarified, while EZH2 overexpression is supposed to be related to downregulation of oncogenes or oncosuppressors. Furthermore, EZH2 interacts with β-catenin, menin, and Wnt signaling pathway suppressors (11). EZH2 represses growth-suppressive Axin2 and negatively regulates β-catenin (14).

ZFX gene encodes a transcription factor related to cell renewal maintenance. Somatic mutations have been identified in 4.6% in abnormal parathyroid tissue, while gene oncogenesis mechanism remains unclear (11).

Wnt/β-catenin/CTNNB1 signaling pathway is enrolled in tumorigenesis, but its role in parathyroid adenoma has not been yet elucidated. β-catenin is the first molecule in Wnt pathway. The Costa-Guda et al. study in 97 patients with sporadic parathyroid adenoma for mutations in exon 3 on CTNNB1 gene was negative for novel mutations except for S37A mutation which is already known from previous studies (18). S37A mutation in exon 3 of CTNNB1 gene has been detected in 7.3% of sporadic adenomas and is related to β-catenin, while its functional role has been verified by immunohistochemical examination, in which β-catenin was found overexpressed in abnormal tissue (19). Furthermore, another somatic mutation S33C in exon 3 of CTNBB1 gene has been detected by Guarnieri et al. after gene sequencing, whereas cyclin D1 and β-catenin expression has not been found altered in immunohistochemical examination (20). Starker et al. restudied somatic mutations in exon 3 and resulted that these mutations are rarely detected in parathyroid adenoma (0.68% for S33C), while in an immunohistochemical study β-catenin was found elevated in mutated cells (21). Other factors related to both Wnt signaling pathway and parathyroid adenoma pathogenesis are the following: (a) LRP5 gene, which is associated with β-catenin destruction complex and is found in 86% in parathyroid adenomas, (b) APC gene, methylated in parathyroid adenomas and (c) RASSF1A gene, which is hypermethylated and underexpressed in parathyroid adenomas in 71% (11, 14). APC gene mutations were detected in the Park et al. Next-generation sequencing study (22).

Erb/ηer-1/EGFR gene is associated with other endocrine disorders or tumors such as breast cancer. DNA from 33 patients with PHPT was assessed using real-time PCR concluding in one amplification of ErbB-1/Her-1/EGFR gene, two deletions of ErbB-2/Her-2 gene, and six deletions of ErbB-4/Her-4 gene, which was reasonably selected as the most appropriate for further investigation (23).

SMAD3 gene is located in chromosome 15 and encodes a TGF-β pathway molecule that binds to menin. Due to this association with menin, SMAD3 mutations leading to abnormal development of parathyroid chief cells were carefully investigated. Indeed, loss of heterozygosity was detected in 24% of parathyroid adenomas, but SMAD3 cannot be characterized as oncosuppressor gene as clonal mutations were not found (24).

CYP27B1 gene was analyzed for mutations correlated to parathyroid adenoma, as this gene encodes 25-hydroxy-vitamin D-1α-hydroxylase, a P450 mitochondrial enzyme, which converts 25-hydroxy-vitamin D to 1.25-dihydroxy-vitamin D, the functional form. No mutations were detected in the CYP27B1 gene, so its role in parathyroid adenoma pathogenesis was not confirmed (25).

Genetic alterations were detected on chromosome 9 in 10/14 patients with parathyroid adenoma in the Garcia et al. study. These alterations include insertions in 9p22-24 and 9q34, in Xq26 (in 6/14 patients), and in 4q21-28 and 8p22-23 (in 4/14 patients). Deletions were found in chromosomes 11 and 20 (20q12-13) in 2/14 cases (26).

Furthermore, mutations in the ASXL3 gene, two somatic mutations in CDC73 parafibromin gene (Y54X), and one somatic mutation in the EZH2 gene were detected in a Chinese population study. In this study, whole genome sequencing was applied in 22 blood samples from patients with sporadic parathyroid adenoma (27).

A germline mutation (deletion) in CDC73/HRPT2 gene was verified in 35% of patients with sporadic parathyroid adenoma by real-time PCR. This gene is involved in Hyperparathyroidism-jaw tumor, so it is recommended as a possible oncogene in sporadic parathyroid adenoma cases (28). CDC73/HRPT2 gene is rarely involved in sporadic disease, while its mutant allele, caused by germline mutations, is detected in cystic and large adenomas and in recurrent cases (11). Germline mutation in the GCM2 gene was found in patients with sporadic disease threefold more frequent compared to the control group, concluding that patients with this mutation have predisposition in parathyroid adenoma development (29). The GCM2 or GCMB gene encodes an important transcription factor for parathyroid adenomas, while familiar absence causes hypoparathyroidism. Mutations in adenoma cases have been detected as Y282D in predisposed allele, V382M, and Y394S. Oncogenesis mechanism has not been found yet (11). GCM2 was verified to be part of parathyroid adenoma pathogenesis in a next-generation sequencing study by Park et al. (22).

By comparative genomic hybridization (CGH) application, genome from 53 parathyroid adenomas was analyzed, and both insertions in 16p and 19p genes and deletions in 11p and 11q genes were detected. These findings introduce new oncogenes and oncosuppressor genes involved in parathyroid adenoma pathogenesis (30).

By PCR, macroarray, protein, and mRNA expression techniques, additional genes in parathyroid adenoma tissues were inserted in an involved gene list: APOLLON, BCL2, CK19, CK18, CHROMOGRANIN-A, BAX, PARATHORMONE, ATM,MDM2, CK8, CYCLIN D1, SYNAPTOPHYSIN, FLIP και TRAIL (31). Epigenetic alterations have been found in APC, SFRP1 SFRP2, SFRP4, WT1, RIZ1, HIC1, c-Met, and MYC και CDH1 genes (10, 32).

In a review by Westin et al. in 2016, β-catenin is an essential part of molecular pathways in parathyroid adenoma pathogenesis. MEN1, HRPT2, EZH2, RASSF1A, HIC1, WT1, APC, SFRP1, SFRP2, SFRP4, SFRP5, and LRP5 genes are related to β-catenin by promoting or inhibiting its production. Wnt/β-catenin genetic pathway is suggested as the main pathway in sporadic parathyroid adenoma development, although further studies is required to verify its role (14).

Further molecular pathways involved in parathyroid adenoma pathogenesis include angiogenic factors. VEGF, FGF, TGFβ, and IGF1 factors were found to be correlated with parathyroid adenoma. VEGF has a pre-angiogenic action and takes part in cell proliferation. FGF factor is increased in adenomas, as it regulates cell cycle and cell development. TGFβ is also increased in adenomas, related to several cell processes. IGF1 is involved in cell proliferation and stands for the main mediatory factor in PTH anabolic role on bones (33).

Apoptosis factors also seem to be associated with parathyroid adenomas. These molecular pathways include anti-apoptotic and apoptotic agents, of which action is altered in adenoma development. TRAIL protein is bound to DR4 and DR5 death receptors and has an anti-cancer role; although TRAIL protein can create complexes with caspase-8 and FADD, leading to opposite action. TRAIL protein is highly expressed in parathyroid adenomas. FAS protein is a death receptor participating in DISC (death inducing signaling complex) formation and its production is elevated in adenomas. BCL2 gene promotes caspase activation and mitochondrial external membrane permeability and was found decreased in adenomas. MDM2, negative regulator of p53, is elevated in adenomas and acts as oncogene (33).

Lu et al. in 2018 studied genetic factors involved in parathyroid adenoma pathogenesis applying immunohistochemistry, real-time PCR, and spectrometry in both histological types of parathyroid adenomas (oxyphil and chief cell adenomas). CaSR, VDR, PTH, MEN1, FGFR1, CNND1, CDKN1B, GCM2, CYP27B1, CYP24A1, PTHLH, and NDUFA13 expression has no statistical significant difference between two groups. VDR and PTH gene expression in parathyroid adenomas ranges from very low to high compared to normal tissue where PTH and VDR are highly expressed (34).

Although several genes have been detected in sporadic parathyroid adenoma, genetic testing in sporadic disease is not recommended, as the majority of mutations is somatic and is developed after disease progression (13). On the other hand, calcium sensing receptor has been proposed as imaging agent for parathyroid detection (35).

### 3.2 Proteomics

In international literature, studies of proteomic analysis of parathyroid adenoma tissues have been reported. Proteins differently expressed in the abnormal tissues have been measured by Western Blotting, MALDI/TOF, MS spectrometry, and immunohistochemistry. There are a vast number of proteins which expression is modified in parathyroid adenomas. Except for the quantitative measurement of these proteins, studies also focus on their functional role and their localization in cells, in order that genetic pathways related to these proteins are detected. **Table 2** shows the large list of proteins associated with parathyroid adenoma.

**Table 2:** Genomics of parathyroid adenoma development.

Gene	Genetic pathway	Intra- or Extracellular pathway
CaSR	Phospholipase C	Intracellular
MEN1	Wnt/β-catenin	Intracellular
CCND1/PRAD1	Cyclin D1	Intracellular
CDKI	Cyclin D1	Intracellular
ZFX	Cyclin D1	Unknown
CTNNB1	Wnt/β-catenin	Intracellular
EZH2	Wnt/β-catenin	Unknown
POT1	Unknown	Unknown
LRP5	Wnt/β-catenin	Intracellular
APC	Wnt/β-catenin	Unknown
RASSF1A	Wnt/β-catenin	Unknown
Erb/ηer-1/EGFR	Unknown	Unknown
SMAD3	TGF-β	Intracellular
ASXL3	Unknown	Unknown
CDC73/HRPT2	Unknown	Unknown
GCM2	Unknown	Intracellular
APOLLON	Unknown	Unknown
BCL2	Apoptosis	Intracellular
CK19	Unknown	Unknown
CK18	Unknown	Unknown
CHROMOGRANIN A	Unknown	Unknown
BAX	Unknown	Unknown
PTH	Unknown	Unknown
ATM	Unknown	Unknown
MDM2	Apoptosis	Intracellular
CK8	Unknown	Unknown
SYNAPTOPHYSIN	Unknown	Unknown
FLIP	Unknown	Unknown
TRAIL	apoptosis	Extracellular
SFRP1 and SFRP2 and SFRP4	Wnt/β-catenin	Unknown
WT1	Wnt/β-catenin	Unknown
RIZ1	Unknown	Unknown
HIC1	Wnt/β-catenin	Unknown
c-MET	Unknown	Unknown
MYC	Unknown	Unknown
CDH1	Unknown	Unknown
VEGF	VEGF	Intracellular
FGF	VEGF	Intracellular
TGFβ	VEGF	Intracellular
IGF1	VEGF	Intracellular
FAS	Apoptosis/ DISC	Extracellular
VDR	Wnt/β-catenin (?)	Intracellular
CYP27B1 and CYP24A1	Unknown	Unknown
NDUFA13	Unknown	Unknown

The functional role of proteins usually is translated as parts of cell metabolism, cytoskeleton structural stability, cell oxidative stress regulation, cell death, transcription, translation, cell connection, and cell signaling transmission (34, 36–39). Arya et al. detected 206 proteins in parathyroid adenomas with different expression, from which 39 were cytoplasmatic proteins, 48 were attached to cytoplasmatic membrane, 49 were macromolecular complexes, 38 were part of a cellular organelles, 21 were related to cell connection, and 10 have extracellular localization. In particular, from those related to cellular organelles, 19 were found in the cellular nucleus, 9 in the cytoskeleton, 5 in the cytoplasm, 3 in the mitochondria, and 2 in the cytoplasmatic membrane. In terms of their functional role, 37.8% had catalytic function on enzymes, 32% were related to cellular connection, while other functions included cellular processes, metabolism, development, localization, regulation, and stimuli response (36). Same results had also Varshney et al. study, which concluded that 33% of proteins were related to cell regulation, 27% to cell death, 27% to transcription, 13% to cell cycle, and 7% to cell signaling transmission. Furthermore, in this study 11 proteins were detected to be differently expressed in parathyroid adenoma cells compared to normal tissue (37).

In sporadic parathyroid adenomas, localization of several proteins is in the cytoplasm, as Akpinar et al. reported (39). Different proteins are expressed in oxyphil cell adenomas in comparison with chief cell adenomas. Lu et al. reported 199 proteins that are differently expressed between two types of parathyroid adenomas. Compared to normal tissue, 83 proteins seem to have modified expression in chief cell adenomas and 44 proteins in oxyphil cell adenomas (34). Chief cells can be transformed to oxyphil cells during puberty and are recognized by intense appearance of mitochondria in them. In the Akpinar et al. study, several mitochondrial proteins were detected in hyperplastic cells, which can be explained by the cell transformation model described above (39).

ANXA-2, ATP5H, and LMNA are examples of proteins detected in parathyroid adenomas, fact that has been verified by three different studies (37, 39, 40). These three proteins are bound to ubiquitin C, the main molecule of their function (39). Calcium-binding proteins are involved in phospholipase C signaling pathway (PLC) and are overexpressed in parathyroid adenomas (36). Additionally, MAPK, Ras, and IP signaling pathways seem to be related to parathyroid adenomas pathogenesis, as several proteins-parts of these pathways have modified expression in parathyroid adenomas (36). Increased intracellular calcium activates mitochondrial function and oncogenetic and oncosuppressive processes are modified, so it may play an important role in oncogenesis. Chromatic regulatory proteins seem to be underexpressed in adenomas, while mitochondrial proteins are increased (36).

VDAC1, ANXA5, ANXA2, and S100-A11 proteins are increased in parathyroid adenomas. VDAC1 is involved in the mitochondrial molecule release process that intensifies apoptosis, and its overexpression in adenomas is found to cause PTH release to peripheral circulation. Annexin role in oncogenesis is under intense investigation. ANXA-5 is found to be related to apoptotic processes, while ANXA-2 enhances tumor metastasis by reinforcing cell detachment and translocation.

COX-5A, PARK-7, PRDX-3, HSP90, HSP60, and CCT5 proteins are also found overexpressed in parathyroid adenomas. These proteins are involved in early cell response to oxidative stress, fact that characterizes adenomas which have intense cell proliferation and neoangiogenesis. BSPRY protein, related to calcium receptors, is underexpressed in parathyroid adenomas, leading to decreased inhibitory role on calcium receptors. Its role remains unclear (40). Prolactin receptor (PRLr) has been found increased in parathyroid adenoma cell surface, fact that implies prolactin role in parathormone regulation and adenoma pathogenesis (41). Vitamin D receptor (VDR) has been found decreased in parathyroid adenomas tissue, which is verified by genomic analysis as previously mentioned (37).

Donaldio et al., in their proteomic analysis, used paraffin embed parathyroid adenoma tissues which were reconstituted with formalin solution. Ten from 20 proteins detected in their study were found in these tissues, which is of great importance for future studies on specially processed adenoma samples in order that new biomarkers are discovered (38).

## 4 Conclusion

This review gives a detailed analysis of all reported data on genomics and proteomics of parathyroid adenoma. Different signaling pathways are involved in adenoma development. Wnt/β-catenin, MAPK, Ras, IP, TGF-β, and PLC have already been established, including many genes and proteins with different functional roles. Further studies should be applied on understanding parathyroid adenoma pathogenesis and introducing new biomarkers for early histological biomarkers to predict recurrence in other parathyroid glands.

## Author contributions

ACho, TP, AChe, ACha: Conceptualization. ACha, CA, KB: Data curation. ACho, AChe, ACha: Formal analysis. SM, TZ, ACho, CA, KB, KD: Investigation. ACho: Methodology. TP, AChe, ACha: Supervision. TP: Validation. ACho: Writing - original draft. TP, AChe, ACha: Writing - review and editing. All authors contributed to the article and approved the submitted version.
